# Low Carbon Concentration Feeding Improves Medium-Chain-Length Polyhydroxyalkanoate Production in *Escherichia coli* Strains With Defective β-Oxidation

**DOI:** 10.3389/fbioe.2018.00178

**Published:** 2018-11-30

**Authors:** Fakhrul Ikhma Mohd Fadzil, Shoji Mizuno, Ayaka Hiroe, Christopher T. Nomura, Takeharu Tsuge

**Affiliations:** ^1^Department of Materials Science and Engineering, School of Materials and Chemical Technology, Tokyo Institute of Technology, Yokohama, Japan; ^2^Department of Chemistry for Life Sciences and Agriculture, Faculty of Life Sciences, Tokyo University of Agriculture, Tokyo, Japan; ^3^Department of Chemistry, College of Environmental Science and Forestry, State University of New York, New York, NY, United States

**Keywords:** MCL-PHA, fatty acid, carbon feeding, near homopolymer, *Escherichia coli*

## Abstract

Medium-chain-length (MCL) polyhydroxyalkanoates (PHAs) of near homopolymeric composition are unnatural polymers, having almost identical repeating units throughout the polymer chain. These homopolymeric PHAs can be produced by β-oxidation defective bacterial hosts. *Escherichia coli* is an attractive workhorse for the production of such genetically engineered PHAs; however, achieving efficient production of the near homopolymers by β-oxidation defective strains is a major challenge because of a lack of process development studies. In order to address this issue, we investigated the optimization of carbon feeding for efficient production of MCL-PHAs by an *E. coli* strain with defective β-oxidation, LSBJ. Engineered bacteria were cultured in shake-flasks with intermittent feeding of a fatty acid substrate [either decanoate (C10) or dodecanoate (C12)] at various concentrations together with a co-carbon source (glucose, glycerol, or xylose) in order to support cell growth. It was found that feeding low concentrations of both fatty acids and co-carbon sources led to an enhanced production of MCL-PHAs. Additionally, the supplementation of yeast extract improved cell growth, resulting in achieving higher titers of MCL-PHA. As a result, poly(3-hydroxydecanoate) [P(3HD)] and poly(3-hydroxydodecanoate) [P(3HDD)] were produced up to 5.44 g/L and 3.50 g/L, respectively, as near homopolymers by employing the developed feeding strategy. To the best of our knowledge, we record the highest titer of P(3HD) ever reported so far.

## Introduction

Increased concerns for sustainability and eco-friendliness have led to greater interest in the development of green materials as a forward-looking solution for overcoming problems related to the disposal of fossil fuel-derived plastics. Because of the problems associated with non-biodegradable, petroleum-based plastics, biodegradable plastics such as polyhydroxyalkanoates (PHAs) are seen as promising substitutes for these conventional plastics in the current market (Gülsah et al., 2017; Koller, 2018). PHAs are biopolyesters synthesized by a variety of microorganisms as intracellular energy storage molecules that are accumulated under stress conditions (Khanna and Srivastava, 2005; Keshavarz and Roy, 2010). As naturally occurring biopolymers, PHAs have advantages over several types of typical polymers like polypropylene because of their biodegradability, biocompatibility, and renewability (Lu et al., 2009).

PHAs can be classified into three categories depending on the length of the carbon chain; short-chain-length polyhydroxyalkanoates (SCL-PHAs), medium-chain-length polyhydroxyalkanoates (MCL-PHAs), and long-chain-length polyhydroxyalkanoates (LCL-PHAs) (Lee, 1996a; Simon-colin et al., 2008). MCL-PHA copolymers consist of 6–14 carbons in their monomer units, which are mainly synthesized by *Pseudomonas* species (Rai et al., 2011; Gao et al., 2016). Although there have been many studies examining the properties of MCL-PHA copolymers (Ren et al., 2000; Ouyang et al., 2007; Jiang et al., 2012), there have been relatively few studies applied toward the biosynthesis of MCL-PHA homopolymers or near homopolymers in the last decade (Liu et al., 2011; Tappel et al., 2012). Unlike MCL-PHA copolymers, MCL-PHA homopolymers such as poly(3-hydroxydecanoate) [P(3HD)] and poly(3-hydroxydodecanoate) [P(3HDD)] exhibit high crystallinities with melting points around 70–82°C (Chung et al., 2011; Abe et al., 2012; Wang et al., 2017). In addition, MCL-PHA homopolymers are also very flexible with high optical transparency (Hiroe et al., 2016); thus, they can be developed as stretchable films for new biomaterial applications.

However, MCL-PHA homopolymers are not naturally occurring PHAs. Gene modification is required to construct the artificial biosynthetic pathway in bacteria. Using synthetic biology, near homopolymers can be biosynthesized from renewable resources by using an engineered-bacteria-based production platform that allows control of the uniformity of MCL-PHAs monomer composition (Tappel et al., 2012). Previously, an *E. coli* strain with a defective fatty acid β-oxidation pathway, strain CAG18496 (*fadA-* and *fadB-*deletion mutant), was used for the biosynthesis of MCL-PHAs from *trans*-2-alkenoic acids (Sato et al., 2012). The biosynthetic pathway was complemented with the heterologous expression of PHA biosynthetic enzymes from *Pseudomonas* strains and resulted in the production of near homopolymers containing 6–12 carbons. The amount of PHA production acquired from P(3HD) was up to 0.14 g/L containing 99.6 mol% of the dominant monomer unit (see Table 4). Later, Tappel et al. found a strategy to control the SCL- and MCL-PHA monomer repeating unit by deleting two genes (*fadA* and *fadB*) involved in β-oxidation in *E. coli* LS5218 [*fadR601, atoC*(Con)], later known as the *E. coli* LSBJ strain. Tailor-made MCL-PHAs were reported to be produced directly from fatty acids channeled to the PHA biosynthetic pathway expressing PhaJ4_Pp_ and PhaC1_Ps_(STQK), producing the MCL-PHA with an equivalent chain length. The production system designed with direct conversion of MCL-PHAs from related fatty acids accumulated 0.26 g/L of P(3HD) (Tappel et al., 2012). Thereafter, P(3HD) production was improved up to 0.60 g/L by removing the regulatory gene *arcA* from the LSBJ strain (Scheel et al., 2016). Following a recent study, we further enhanced the production of the near P(3HD) homopolymer to 1.47 g/L after growing cells in a mineral-based medium supplemented with 5 g/L yeast extract (Hiroe et al., 2016). However, all the MCL-PHAs production strategies developed so far are still inefficient with lower yields obtained because of the toxicity of fatty acids fed to *E. coli*.

In this study, we developed a feeding strategy for producing MCL-PHAs from fatty acids as direct precursors for PHA biosynthesis and the utilization of co-carbon sources to support bacterial cell growth. The intermittent feeding process was executed by maintaining both fatty acid and co-carbon sources at low concentrations, thereby reducing catabolite repression and toxicity toward *E. coli* cells. The optimization of carbon feeding and supplementation of yeast extract allowed us to improve the conversion yield of fatty acids to the corresponding PHA.

## Materials and methods

### Strains and plasmids

**Figure 1:**
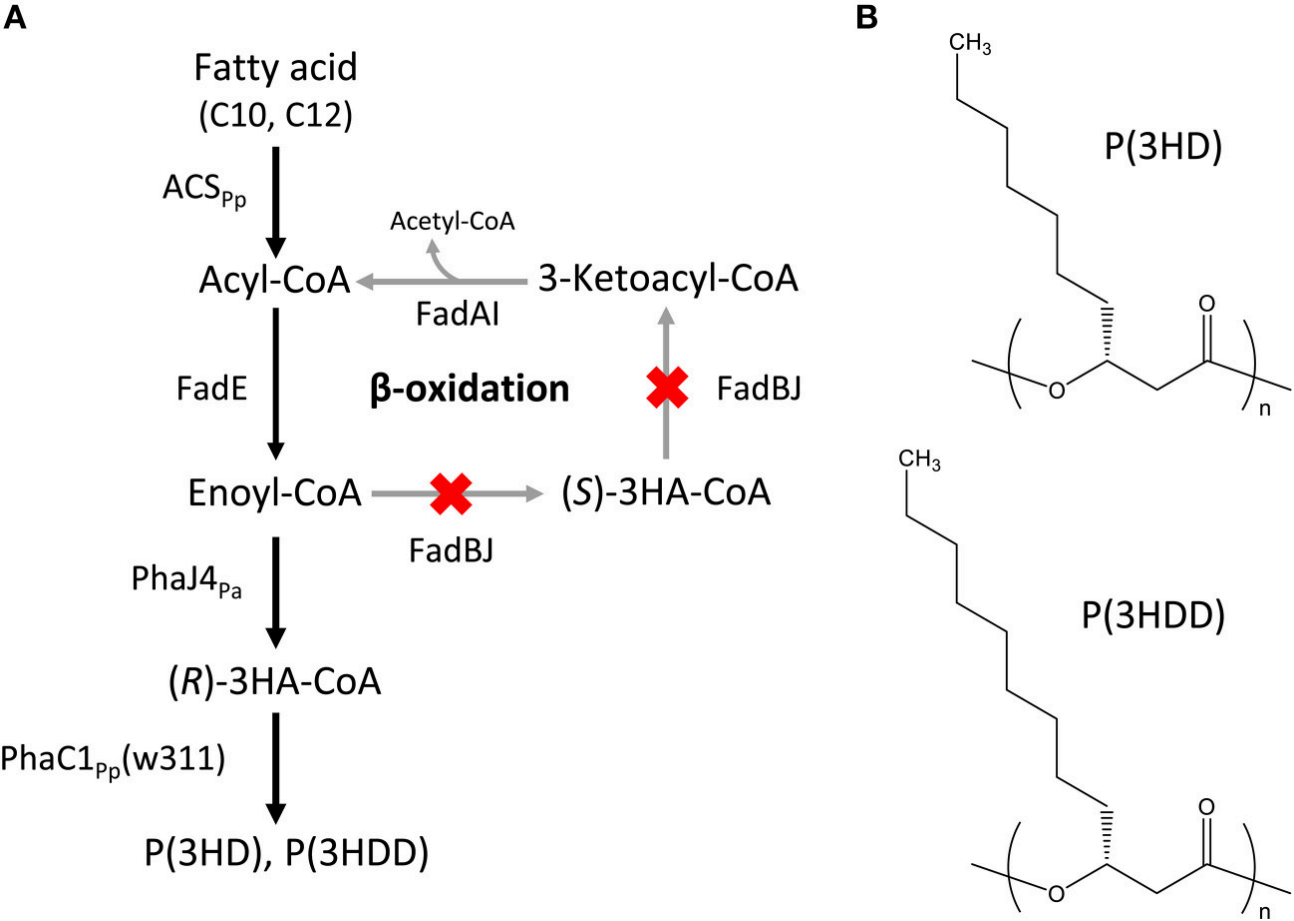
**(A)** Biosynthesis pathway of P(3HD) and P(3HDD). PhaC1_Pp_(w311): PHA synthase 1 from *Pseudomonas putida* KT2440 with two site-specific mutations, Glu358 → Gly (E358G) and Asn398 → Ser (N398S). PhaJ4_Pa_: (*R*)-specific enoyl-CoA hydratase from *P. aeruginosa*, ACS_Pp_: acyl-CoA synthetase from *P. putida* KT2440. **(B)** Chemical structures of P(3HD) and P(3HDD).

The *Escherichia coli* LSBJ strain with *fadB*- and *fadJ*-deletion was used as a host for producing MCL-PHAs (Tappel et al., 2012). The expression vectors introduced into the host were as follows: pBBR1C1_Pp_(w311)J4_Pa_ harboring the evolved PHA synthase 1 gene (*phaC1*_Pp_) from *P. putida* KT2440 with two site-specific mutations, Glu358 → Gly (E358G) and Asn398 → Ser (N398S) (w311 mutant, Hiroe et al., 2018), together with *R*-specific enoyl-CoA hydratase 4 gene (*phaJ4*_Pa_) from *P. aeruginosa* (Tsuge et al., 2003); and pTTQACS_Pp_, which had been constructed previously, carrying the acyl-CoA synthetase gene (*acs*_Pp_) from *P. putida* KT2440 (Hiroe et al., 2016). The constructed pathway leading to the biosynthesis of P(3HD) and P(3HDD) is shown in Figure 1.

### Culture conditions

Expression vectors were transformed into *E. coli* LSBJ, and transformant cultures were grown at 37°C in a reciprocal shaking incubator for 16 h in Lysogeny-Broth (LB) medium supplemented with 50 mg/L kanamycin and 50 mg/L carbenicillin. The LB medium contained (per liter) 10 g NaCl, 10 g tryptone (Difco, Detroit, MI, USA), and 5 g yeast extract (Difco). The overnight cultures were transferred to shake flasks containing the same antibiotics and supplemented with glucose, glycerol, or xylose as a co-carbon source. Unless otherwise stated, the initial co-carbon sources for cultures with fed-batch feeding were set at 1 g/L. All cultivations were carried out in MR medium (Kahar et al., 2005) containing (per liter) KH_2_PO_4_, 13.5 g; (NH_4_)_2_HPO_4_, 4 g; citric acid, 1.7 g; MgSO_4_·7H_2_O, 1.4 g; thiamine, 10 mg/L; and trace metal solution, 10 mL. The trace metal solution consisted of the following (per liter of 0.1 M HCl): FeSO_4_·7H_2_O, 10.0 g; CaCl_2_, 2.0 g; ZnSO_4_·7H_2_O, 2.2 g; MnSO_4_·4H_2_O 0.5 g; CuSO_4_·5H_2_O, 1.0 g; (NH_4_)_6_Mo_7_O_24_·4H_2_O, 0.1 g; and Na_2_B_4_O_7_·10H_2_O, 0.02 g. The pH of the MR medium was adjusted to 7.0 by KOH. The MRY medium was prepared by replacing thiamine in the MR medium with 20 g/L yeast extract.

Subsequent feeding with a co-carbon source was started after 12 h and was followed by intermittent fed-batch feeding for every 8 h until 84 h. Overexpression of *acs*_Pp_ was induced by the addition of 1 mM isopropyl-β-D-thiogalactopyranoside (IPTG) at 12 h. Sodium decanoate (C10Na) or sodium dodecanoate (C12Na) was intermittently added as PHA precursors in the same manner as the carbon feedstocks for growth. When using C12Na as a carbon source, the chemical surfactant, BRIJ35 (Wako Chemical, Osaka, Japan) was added to a final concentration of 0.4 v/v% in order to increase the solubility of fatty acids in the medium. Incubation of shake flasks for PHA production was performed in a reciprocal shaker at 30°C and 130 rpm for 96 h.

### Analytical procedures

The dry cell weight was gravimetrically measured after centrifuging the culture medium at 6,000 × *g* for 15 min at 4°C, washed with water/hexane, and lyophilized for 3 days. PHA content, PHA concentration, and 3HA monomer compositional analysis were determined by gas chromatograph (GC), using Shimadzu GC-2014s (Shimadzu, Kyoto, Japan) with a flame-ionization detector. Dried cells were subjected to methanolysis to convert 3HAs into the 3HA-methyl ester constituents. After heating at 100°C for 140 min, the reaction mixture was cooled to room temperature and 1 mL of distilled water was added to separate the polar from non-polar components. The non-polar components, containing 3HA-methyl esters, was filtered and mixed with 0.5 mL of the internal standard solution containing methyl-*n*-octanoate in 0.1% (w/v) chloroform. Samples were automatically injected through the GC capillary column, Neutra Bond-1 (30 m × 0.25 mm, GL Science, Tokyo, Japan). The column temperature was initially set at 120°C and then increased to 280°C at a rate of 8°C/min. The signal-peak areas obtained were calculated for total PHA content and 3HA monomers. The molecular weight of MCL-PHAs was measured by gel permeation chromatograph (GPC) using a Shimadzu 10A GPC system with 10A refractive index detector equipped with Shodex K-806M and K-802 columns at 40°C. Chloroform was used as the eluent at a flow rate of 0.8 mL/min. The sample concentration and injection volume were set at 1 mg/mL and 100 μL, respectively. To construct the calibration curve, polystyrene standards with low polydispersity were used.

## Results

### Effect of carbon substrate and feedstock concentrations on MCL-PHA production

**Figure 2:**
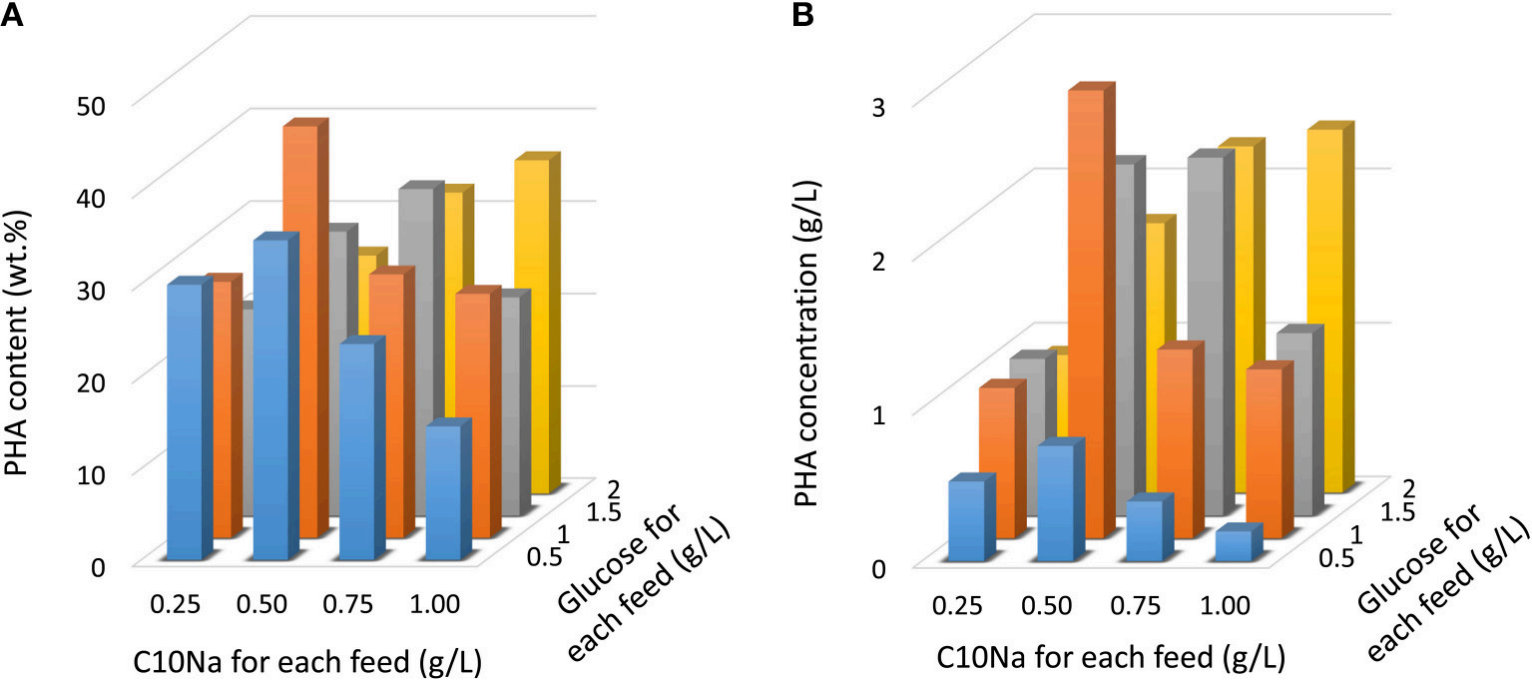
PHA production in *E. coli* LSBJ harboring pBBR1C1_Pp_(w311)J4_Pa_ and pTTQACS_Pp_ with glucose (0.5–2 g/L) and C10Na fatty acid (0.25–1 g/L) feeding. Cells were cultivated at 30°C for 96 h in the MR medium initially containing glucose (0.5–2 g/L) with fatty acid/glucose addition beginning at 12 h. Subsequent carbons were fed 10 times at 8 h intervals. **(A)** PHA content. **(B)** PHA concentration.

Since the *E. coli* strain LSBJ is unable to grow on fatty acids as a feedstock due to the break in β-oxidation pathway, and instead uses these fatty acids as a substrate to generate PHA, a co-carbon feedstock such as glucose was necessary to support cell growth. To assess the effect of dual-carbon source utilization in MCL-PHA production, cells were grown in the MR medium with glucose (0.5–2 g/L) and fatty acid (0.25–1 g/L) feeding for 10 times. Figure 2 shows the results obtained by intermittent fed-batch cultures after 96 h of cultivation. By employing this strategy, the highest PHA content was obtained with C10Na and glucose feeding at the concentrations of 0.5 and 1 g/L, respectively, resulting in a PHA content of 44.6 wt.%. Using this feeding combination, 2.91 g/L PHA was achieved. When the C10Na feeding was slightly increased to 0.75 g/L, PHA content was reduced to 28.6 wt.%. and the PHA concentration was decreased to 1.23 g/L. A further increase in C10Na feeding to 1.0 g/L showed an additional decrease in PHA content and PHA concentration to 26.5 wt.% and 1.10 g/L, respectively. When the C10Na feeding was reduced to 0.25 g/L, PHA content was obtained at 27.8 wt.%, accounted for 0.98 g/L PHA. A similar trend was observed for 0.5 g/L glucose feeding; a PHA content of 14.6 wt.% was attained when co-fed with 1 g/L C10Na. Despite all this, further increases in the fatty acid feeding concentration increased the PHA production with 2.0 g/L glucose feeding.

**Figure 3:**
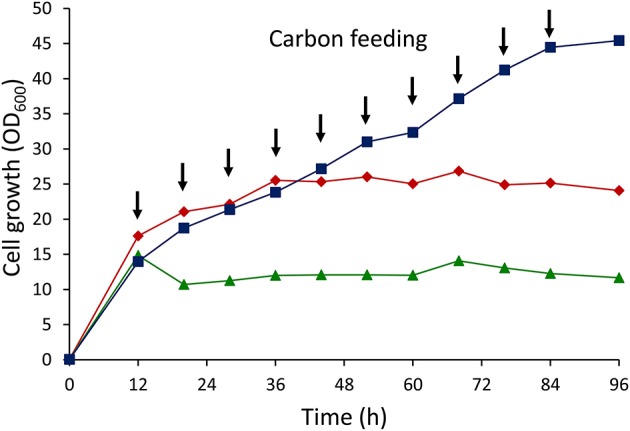
Cell growth (OD_600_) curves of *E. coli* LSBJ harboring pBBR1C1_Pp_(w311)J4_Pa_ and pTTQACS_Pp_ using different glucose feeding strategies. (Squares) 1 g/L glucose intermittent feeding for 10 times together with 0.5 g/L C10Na, (diamonds) 5 g/L glucose batch addition with feeding 0.5 g/L C10Na for 10 times, (triangles) 10 g/L glucose batch addition with feeding 0.5 g/L C10Na for 10 times. Arrow points indicated the addition times of glucose (1 g/L) + C10Na (0.5 g/L) or C10Na (0.5 g/L) only.

The results of cell growth experiments with different glucose feeding strategies are shown in Figure 3. Cell growth remained stagnant after 36 h under growth conditions where 5 g/L glucose was fed by batch addition. Further increase in the glucose concentration up to 10 g/L retarded the cell growth, with dramatic decreases in growth after just 12 h. However, cell growth sustained throughout the cultivation by applying intermittent addition of glucose. This result clearly demonstrated that dual-carbon feeding at low concentrations was crucial for maintaining the cell growth.

Based on these results, the condition for achieving the highest PHA content was selected for our standard cultivation condition (feeding of 0.5 g/L fatty acid and 1.0 g/L co-carbon source) and was used in the subsequent study.

### Effect of co-carbon source on MCL-PHA production

A wide variety of carbon sources can be used to support cell growth of *E. coli*. Here, we assessed the utilization of co-carbon sources other than glucose, notably glycerol and xylose, for cell growth to produce P(3HD). The optimal concentration for glucose was also suitable for glycerol and xylose in accordance with those dry cell weights obtained as previously reported (Mizuno et al., 2018). Additionally, the usability of a longer chain fatty acid, C12Na, was evaluated for P(3HDD) production. The production yields for P(3HD) and P(3HDD) are shown in Table 1. Cultivation with glucose resulted in a markedly lower P(3HD) concentration (2.91 g/L) than that with other co-carbon sources, despite being the preferred carbon source for *E. coli*. On the other hand, the highest P(3HD) concentration (4.21 g/L) was achieved when cells were grown with glycerol, which is a less favorable carbon source for *E. coli*. Cultivation with xylose showed the same features as glycerol, surpassing the PHA concentration obtained from glucose. Subsequently, we also tested the use of C12Na to expand the biosynthesis range of the MCL-PHAs. When C12Na was fed to the culture medium, cell growth was decreased compared to cultivations with C10Na. The highest P(3HDD) production of 3.11 g/L was obtained with glycerol.

**Table 1:** PHA production by *E. coli* LSBJ harboring pBBR1C1_Pp_(w311)J4_Pa_ and pTTQACS_Pp_ in MR medium with feeding fatty acid (0.5 g/L) and co-carbon source (1 g/L) for 10 times.

**Feeding (**× **10 times)**		**Dry cell wt. (g/L)**	**PHA content (wt.%)**	**PHA conc. (g/L)**	**Yield (mol-3HA/mol-fatty acid)**	**PHA composition (mol%)** ^**a**^			**Molecular weight**	
**Co-carbon source (1 g/L each)**	**Fatty acid (0.5 g/L each)**					**3HO**	**3HD**	**3HDD**	***M_*w*_*(× 10^5^)**	***M_*w*_*/*M_*n*_***
Glucose	C10Na	6.52 ± 0.06	44.6 ± 1.0	2.91 ± 0.04	0.61	0.3	99.7	–	2.03	3.3
	C12Na	4.47 ± 0.08	45.0 ± 1.4	2.01 ± 0.03	0.42	0.1	1.4	98.5	1.25	2.2
Glycerol	C10Na	7.33 ± 0.23	57.4 ± 2.2	4.21 ± 0.06	0.88	0.3	99.7	–	1.67	3.9
	C12Na	7.04 ± 0.12	44.2 ± 1.3	3.11 ± 0.07	0.65	0.1	1.0	98.9	1.18	2.3
Xylose	C10Na	6.20 ± 0.16	63.3 ± 3.0	3.92 ± 0.16	0.82	0.2	99.8	–	1.24	2.4
	C12Na	5.50 ± 0.15	52.5 ± 1.1	2.89 ± 0.09	0.60	-	0.6	99.4	1.62	2.4

*Cells were cultivated at 30°C for 96 h in the MR medium initially containing 1 g/L co-carbon sources with fatty acid addition began at 12 h and subsequent carbon feeding were performed every 8 h for 10 times. Results are averages of independent triplicate experiments*.

a *PHA composition and contents in the cells were determined by GC. 3HO, 3-hydroxyoctanoate; 3HD, 3-hydroxydecanoate; 3HDD, 3-hydroxydodecanoate*.

GC analysis also confirmed that the PHAs produced from C10Na contained 3HD monomers close to the homopolymer, in the range of 99.7–99.8 mol%. In some manner, 3HDD retained its major monomer units far less between 98.5 and 99.4 mol%. Minor fraction of 3-hydroxyoctanoate (3HO) was produced due to the presence of homolog enzymes (FadL, FadD, and FadE), responsible for breaking down the supplied fatty acid, thus contributed toward the incorporation of shorter chain monomer. The weight-average molecular weight (*M*_*w*_) of P(3HD) reached the highest at 2.03 × 10^5^ under glucose feeding, whereas the highest for P(3HDD) was obtained with xylose feeding at 1.62 × 10^5^. The effect of a longer fatty acid chain added to *M*_*w*_ was distinctive only with xylose feeding. The polydispersity (*M*_*w*_/*M*_*n*_) of the acquired P(3HD) samples was larger than that of P(3HDD) with glucose and glycerol feeding, but was equal with xylose feeding.

### MCL-PHA production with MRY medium

P(3HD) production was enhanced by limiting the glucose and fatty acid availability. However, as a result, cell mass production was sluggish. Thus, we examined the yeast extract-supplemented medium (MRY medium) for further enhancing PHA production by improving cell mass production. MRY medium was prepared by replacing thiamine in the MR medium with 20 g/L yeast extract, and the culture results are shown in Table 2.

**Table 2:** PHA production by *E. coli* LSBJ harboring pBBR1C1_Pp_(w311)J4_Pa_ and pTTQACS_Pp_ in MRY medium with feeding fatty acid (0.5 g/L) and co-carbon source (1 g/L) for 10 times.

**Feeding (**× **10 times)**		**Dry cell wt. (g/L)**	**PHA content (wt.%)**	**PHA conc. (g/L)**	**Yield (mol-3HA/mol-fatty acid)**	**PHA composition (mol%)** ^**a**^		
**Co-carbon source (1 g/L each)**	**Fatty acid (0.5 g/L each)**					**3HO**	**3HD**	**3HDD**
Glucose	C10Na	9.38 ± 0.01	38.9 ± 1.7	3.65 ± 0.12	0.76	0.6	99.4	–
	C12Na	7.73 ± 0.59	28.1 ± 6.8	2.15 ± 0.42	0.45	1.8	4.1	94.1
Glycerol	C10Na	10.63 ± 0.50	29.5 ± 3.1	3.15 ± 0.48	0.66	1.4	98.6	–
	C12Na	9.51 ± 0.27	32.4 ± 0.8	3.08 ± 0.15	0.64	0.8	3.1	96.1
Xylose	C10Na	8.13 ± 1.44	24.9 ± 6.8	2.07 ± 0.83	0.43	1.4	98.6	–
	C12Na	8.33 ± 0.18	42.0 ± 4.3	3.50 ± 0.28	0.73	0.4	2.1	97.5

*Cells were cultivated at 30°C for 96 h in the MRY medium (MR medium containing 20 g/L yeast extract) initially containing 1 g/L co-carbon source with fatty acid addition began at 12 h and subsequent carbon feeding were performed every 8 h for 10 times. Results are averages of independent triplicate experiments*.

a *PHA composition and contents in the cells were determined by GC. 3HO, 3-hydroxyoctanoate; 3HD, 3-hydroxydecanoate; 3HDD, 3-hydroxydodecanoate*.

MRY medium significantly improved cell mass production for all cultures. The highest cell mass of 10.63 g/L was obtained when the strain was grown with glycerol. Cultivation with glucose improved P(3HD) and P(3HDD) yields to 3.65 and 2.15 g/L, respectively. However, the considerable cell mass production caused a significant reduction in PHA content. Under all conditions, PHA concentrations were generally increased with the only exception being for xylose used to synthesize P(3HD). Adversely, yeast extract supplementation reduced the dominant monomer composition to a moderate extent with notable effects observed in 3HDD production. Notably, glucose feeding resulted in a discernible reduction, a 4.4% decrease in the total 3HDD fraction.

### Increased fatty acid feeding for P(3HD) production

Up to this stage, the effect of co-carbon sources had been elucidated. However, examination of the results obtained in this study show that P(3HD) production with glycerol and xylose as co-fed with fatty acids exhibited very high conversion yields, exceeding 0.8 mol-3HA/mol-fatty acid (Table 1). In these cultures, precursor fatty acids for P(3HD) produced via native metabolism might be deficient, thus maximum levels of P(3HD) production may have been reached under the given conditions. Thus, we next tested a slightly higher C10Na concentration of 0.75 g/L for each feeding, using MR and MRY media. The results are presented in Table 3. Fatty acid fed to the MR medium at 0.75 g/L led to a reduction in cell mass and P(3HD) production under glucose feeding. By adding fatty acid substrate at this concentration, we also discovered that using either glycerol and xylose as a feedstock increased both cell mass and P(3HD) production. In contrast, the MRY medium with decanoate and glucose co-feeding showed an exceptional improvement, leading to the highest P(3HD) production (5.44 g/L). We then evaluated the molecular weight from samples obtained with several culture conditions. By allowing for a higher concentration of fatty acids to be fed to the bacteria, it was found that the molecular weight of P(3HD) samples were slightly reduced with narrower polydispersities (Table 3).

**Table 3:** Increased feeding of C10Na (0.75 g/L each) for P(3HD) production by *E. coli* LSBJ harboring pBBR1C1_Pp_(w311)J4_Pa_ and pTTQACS_Pp_.

**Feeding (× 10 times)**	**Medium**	**Dry cell wt. (g/L)**	**PHA content (wt.%)**	**PHA conc. (g/L)**	**Yield (mol-3HA/mol-fatty acid)**	**PHA composition (mol%)**^**a**^		**Molecular weight**	
						**3HO**	**3HD**	***M_*w*_*(× 10^5^)**	***M_*w*_*/*M_*n*_***
Glucose (1 g/L) + C10Na (0.75 g/L)	MR	4.50 ± 1.30	28.6 ± 7.4	1.23 ± 0.17	0.17	0.3	99.7	–	–
	MRY	10.16 ± 0.58	53.4 ± 0.6	5.44 ± 0.65	0.76	0.4	99.6	1.18	2.9
Glycerol (1 g/L) + C10Na (0.75 g/L)	MR	7.45 ± 0.41	59.9 ± 7.4	4.44 ± 0.34	0.62	0.2	99.8	1.15	2.0
	MRY	9.00 ± 0.62	39.9 ± 5.9	3.58 ± 0.53	0.50	1.3	98.7	–	–
Xylose (1 g/L) + C10Na (0.75 g/L)	MR	7.55 ± 0.15	66.3 ± 4.9	5.00 ± 0.27	0.70	0.2	99.8	1.27	2.0
	MRY	9.80 ± 1.00	45.5 ± 8.2	4.40 ± 0.40	0.61	0.7	99.3	–	–

*Cells were cultivated at 30°C for 96 h in the MR or MRY medium initially containing 1 g/L co-carbon sources with fatty acid addition (0.75 g/L) began at 12 h and subsequent carbon feeding were performed every 8 h for 10 times. Results are averages of independent triplicate experiments*.

a *PHA composition and contents in the cells were determined by GC. 3HO, 3-hydroxyoctanoate; 3HD, 3-hydroxydecanoate; 3HDD, 3-hydroxydodecanoate*.

## Discussion

In this study, efficient conversion of fatty acids to MCL-PHAs was achieved by implementing an intermittent fed-batch co-feeding of dual carbon substrates at low concentrations. The fed-batch feeding approach was implemented to investigate cell mass production and optimize PHA accumulation. The fed-batch feeding strategy was able to maintain exponential cell growth by reducing the inhibitory effects that occurred when cells are fed high concentrations of carbon. Measuring PHA content enabled us to understand the adaptive response toward fatty acid and co-carbon source feeding at different concentration levels. Cells that responded adaptively to conditions mediated by a trade-off balance were able to accumulate high PHA content and sustain high cell mass production. Glucose concentration for each feeding was optimal at 1 g/L, whereas low fatty acid feeding concentrations showed notable effects at 0.5 g/L (Figure 2). The same trend was previously reported as higher concentrations of *trans*-2-decenoic acid added to the mineral medium significantly reduced PHA content by 38% (Sato et al., 2012).

Meanwhile, the PHA production was enhanced when fatty acids were supplied at a higher concentration (increasing from 0.75 to 1.0 g/L) with glucose feeding at 2 g/L. This represents the supportive effect of glucose under higher fatty acid concentrations. Cultures were shown to co-metabolize glucose and C10Na in a balanced state. Although the *E. coli* LSBJ strain possesses a higher tolerance toward fatty acids, up to 2 g/L, cells were still unable to accumulate higher PHA concentration because of a finite fatty acid feeding scheme. Here, the intermittent fed batch feeding was shown to be advantageous to further increase PHA accumulation. The approach increased the amount of fatty acid fed to the culture within the toxicity limits. This may improve the cellular fatty acid uptake, thus contributing to an effective conversion toward MCL-PHAs.

Direct utilization of renewable sugars as a carbon source for fermentation is of great interest for advancing the expandability of the production platform (Mizuno et al., 2018). When PHA accumulation was determined, polymer content was observed to accompany cell growth in all cultures, reaching levels as high as 63.3 wt.%, acquired with xylose feeding (Table 1). We found that although glucose is known for being the most preferred carbon source, cell growth and PHA production were much lower than those obtained from glycerol and xylose. This suggests that the hierarchy of preference for *E. coli* cells follows the least desirable carbon feedstock uptake order when there is more than one carbon source available. As expected, the consequent effect of toxicity was evident when a longer fatty acid chain was used to synthesize P(3HDD). Another possible influence that may also affect PHA accumulation is the role of a strictly regulated *tac* promoter, which is highly effective in controlling the overexpression of ACS_Pp_ under restricted glucose feeding. Despite this, higher levels of MCL-PHA production were observed when xylose was co-fed with C10Na. This finding suggests that a higher PHA conversion may take place under slower co-carbon source uptake.

The effect of a longer fatty acid chain on the molecular weight was distinct when grown with glucose and glycerol (Table 1). The molecular weight analysis also revealed that the use of w311 mutant synthase produced polymers with significantly higher molecular weights than those produced from the parent synthase as previously reported (Hiroe et al., 2018). Hence, the high *M*_*w*_ of MCL-PHAs produced in this study provided significant flexibility and could eventually offer greater extensions in the processing window for these materials and thus the potential for their use in broad applications.

An appropriate supply of nitrogen is important to achieve high cell density cultivation (Lee, 1996b). Yeast extract is considered as one of the major sources not only for carbon but also for nitrogen in bacterial cell growth. Yeast extract added to the basic medium was proven to be effective because it allowed for high-density growth (Shiloach and Fass, 2005). Yeast extract supplementation (MRY medium) could substantially improve cell growth, but had a different effect on PHA accumulation depending on the co-carbon source used (Tables 2, 3). By supplementing with yeast extract, the PHA content was decreased as predicted due to a considerable increase in cell mass. We found that the addition of yeast extract to the medium containing glucose had a profound effect on P(3HD) production. The reason behind this was due to yeast extract supplementation that eventually induced faster cell growth and promoted higher co-carbon utilization. This finding also further convinced us that PHA accumulation strongly depends upon the availability and utilization of the co-carbon source. The reason why P(3HDD) concentration is higher than P(3HD) under cultivation with xylose remains to be elucidated (Table 2).

Fatty acid toxicity toward *E. coli* cells depends on the chain length and feeding concentration. We observed that yeast extract addition played an important role in improving PHA production under higher fatty acid concentration for feeding (Table 3). Under all tested conditions, cells produced the highest amount of MCL-PHA in the MRY medium with glucose as a co-carbon source. This observation implies that yeast extract addition can increase cell tolerance toward fatty acid toxicity. Moreover, P(3HD) production was enhanced in mineral medium with added yeast extract for glycerol and xylose cultures. This exciting observation provides us with an expedient method which offers innovative and efficient production of biopolymers from a number of carbon sources.

Here, our ultimate objective to improve high level MCL-PHA production has been achieved. Understanding the effects of fatty acid feeding and the role of yeast extract has allowed us to develop a robust process for MCL-PHA production. Notably, we have improved the production of MCL-PHAs by intermittent feeding of co-carbon sources and fatty acids at fairly low concentrations. Yeast extract supplementation positively influenced P(3HD) production under slightly higher fatty acid feeding concentrations. Table 4 summarizes the strategies for P(3HD) production reported previously. The first detailed study for achieving a near P(3HD) homopolymer in *E. coli* was performed with batch glucose addition at a moderate concentration (5 g/L) (Sato et al., 2012). Low cell mass concentration was obtained in this work and was thought to be the reason for low polymer production. Similarly, low P(3HD) production was acquired when cultures were grown in a medium without a co-carbon source (Scheel et al., 2016). When glycerol was supplemented as a co-carbon source at 10 g/L, a high mass concentration of 6.04 g/L was reached in accordance with an improvement in the polymer yield (Hiroe et al., 2016). The high cell mass concentration achieved shows an effective carbon uptake, leading to a higher PHA production. In addition, the high cell concentration reached in this work also contributed to higher production of PHA. For this purpose, the importance of maintaining low carbon concentrations was highlighted in this study. The recombinant *E. coli* strain grown was capable of accumulating up to 5.4 g/L P(3HD) containing 99.6 mol% 3HD units, the highest production ever reported to date. Moreover, a high P(3HDD) production of 3.50 g/L was achieved in the same manner by intermittent feeding of xylose as a co-carbon source and C12 fatty acid at fairly low concentrations (0.5 g/L).

**Table 4:** Comparison of strategies for P(3HD) production reported previously.

**Polymer yield (g/L)**	**3HD fraction (mol%)**	**Strategy for P(3HD) production**	**References**
0.143^a^	99.6	C10 unsaturated fatty acid (0.5 g/L) and glucose (5 g/L) were initially supplemented into M9 mineral medium, with *E. coli* CAG18496 (*ΔfadAB*) expressing *acs*_Pp_, *phaJ4*_Pa_, and *phaC1*_Pp_ as a production strain.	Sato et al., 2012
0.603	Nearly 100	C10 fatty acid (2 g/L) was initially supplemented into LB medium, with *E. coli* RSC02 (*ΔfadBJ, ΔarcA*) expressing *phaJ4*_Pp_ and *phaC1*_Ps_(STQK) mutant as a production strain.	Scheel et al., 2016
1.47	99.6	Glycerol (10 g/L) was initially supplemented into MR mineral medium and C10 fatty acid (2 g/L) was supplemented once after 6 h of cultivation, with *E. coli* LSBJ (*ΔfadBJ*) expressing *acs*_Pp_, *phaJ4*_Pa_, and *phaC1*_Pp_ as a production strain.	Hiroe et al., 2016
2.04^a^	99.6	C10 fatty acid (12 g/L) was initially supplemented into T5Y medium (12 g/L tryptone, 24 g/L yeast extract), with *Pseudomonas entomophila* LAC23 (*ΔfadAB, ΔPSEEN0664*) as a production strain.	Wang et al., 2017
5.00	99.8	Xylose (1 g/L) and C10 fatty acid (0.75 g/L) feeding into MR mineral medium was began at 12 h and subsequent feedings were performed every 8 h for 10 times, with *E. coli* LSBJ (*ΔfadBJ*) expressing *acs*_Pp_, *phaJ4*_Pa_, and *phaC1*_Pp_ (w311) mutant as a production strain.	This study
5.44	99.6	Glucose (1 g/L) and C10 fatty acid (0.75 g/L) feeding into MRY medium was began at 12 h and subsequent feedings were performed every 8 h for 10 times, with *E. coli* LSBJ (*ΔfadBJ*) expressing *acs*_Pp_, *phaJ4*_Pa_, and *phaC1*_Pp_ (w311) mutant as a production strain.	This study

a *Polymer yield was calculated from dry cell weight and PHA content*.

## Author contributions

FM, SM, AH, and TT jointly conceived the study. FM and SM performed the experiments, and FM wrote the manuscript in consultation with AH, CN and TT. All authors read and approved the final manuscript.

### Conflict of interest statement

The authors declare that the research was conducted in the absence of any commercial or financial relationships that could be construed as a potential conflict of interest.
